# NT-3-Expressing Olfactory Ensheathing Cells Reduce Brain Aβ1–42 Accumulation and Improve Cognitive Performance in a Rat Model of Alzheimer’s Disease

**DOI:** 10.3390/cells15181706

**Published:** 2026-09-19

**Authors:** Ekaterina Konstantinovna Karsuntseva, Anastasia Denisovna Voronova, Olga Vladislavovna Stepanova, Aleksei Anatolyevich Stepanenko, Valentina Sergeevna Shishkina, Grigorii Andreevich Fursa, Andrey Victorovich Chadin, Igor Vladimirovich Reshetov, Olga Ivanovna Gurina, Vladimir Pavlovich Chekhonin

**Affiliations:** 1 Department of Fundamental and Applied Neurobiology, V.P. Serbsky Federal Medical Research Center for Psychiatry and Narcology, Moscow 119034, Russia; nastyanastyav@mail.ru (A.D.V.); sms-34@yandex.ru (O.V.S.); a.a.stepanenko@gmail.com (A.A.S.); shishkinavalya@mail.ru (V.S.S.); gregorii.fursa@gmail.com (G.A.F.); chadin_777@mail.ru (A.V.C.); olga672@yandex.ru (O.I.G.); chekhoninnew@yandex.ru (V.P.C.); 2 Department of Medical Nanobiotechnology, Pirogov Russian National Research Medical University, Moscow 117513, Russia; 3 University Clinical Hospital No. 1, Sechenov First Moscow State Medical University (Sechenov University), Moscow 119435, Russia; 2487784@mail.ru; 4 Academy of Postgraduate Education, Federal Research and Clinical Center, Federal Medical-Biological Agency, Moscow 125371, Russia; 5 Research Institute of Personalized Medicine, Endocrinology Research Centre, Moscow 117292, Russia

**Keywords:** Alzheimer’s disease, beta-amyloid 1–42, cell-based gene therapy, ensheathing cells from olfactory mucosa, behavioral tests, neurotrophin-3

## Abstract

**Highlights:**

**What are the main findings?**

- Transplantation of both non-transduced and NT-3-transduced olfactory ensheathing cells (OECs) improved behavioral and cognitive performance over the 4-week observation period.
- Only NT-3-transduced OECs reduced brain Aβ1–42 and increased NT-3 concentrations in a rat model of Alzheimer’s disease 4 weeks after transplantation.

**What are the implications of the main findings?**

- NT-3-expressing OECs constitute a promising cell-based gene therapy strategy for Alzheimer’s disease.
- The feasibility of harvesting autologous OECs supports the potential for translational application of this therapeutic strategy.

**Abstract:**

Cell-based gene therapy is a promising strategy to promote regeneration in Alzheimer’s disease (AD). Neurotrophin-3 (NT-3) plays a key role in neuroprotection and brain regeneration, providing a strong rationale for its exogenous delivery. Olfactory ensheathing cells (OECs) have high regenerative potential and serve as a vehicle for local gene delivery. We investigated the therapeutic potential of NT-3-expressing OECs in an Aβ1–42-induced AD model in female Wistar rats. Rat OECs were transduced with an Ad5RGD-CAG-NT-3 adenoviral vector, and NT-3 levels were measured in vitro using ELISA. To evaluate the efficacy of NT-3-transduced and non-transduced OECs, we performed behavioral assessments and measured the concentration of NT-3, Aβ1–42, and pTau levels in brain specimens 4 weeks post-transplantation. Transduced OECs exhibited markedly increased NT-3 secretion in vitro compared with non-transduced cells. All OEC-treated groups showed improved cognitive performance following transplantation. However, only NT-3-transduced OECs significantly increased brain NT-3 and reduced Aβ1–42 levels, indicating an enhanced therapeutic effect mediated by NT-3 overexpression. None of the cell grafts affected pTau levels. These findings demonstrate that OEC transplantation improves functional outcomes in experimental AD, while NT-3 overexpression provides biochemical benefits by reducing amyloid accumulation. Taken together, our findings support further investigation of NT-3-expressing OECs as a cell-based gene therapy for AD.

## 1. Introduction

Alzheimer’s disease (AD) represents a major medical and social challenge and remains one of the leading causes of disability and mortality among individuals over 65 years of age worldwide [1]. Advances in diagnostic techniques are enabling earlier detection of AD, including in younger patients [2]. AD is characterized by the deposition of beta-amyloid (Aβ) as senile plaques and the accumulation of phosphorylated tau protein (pTau) in neurofibrillary tangles within the cerebral cortex, hippocampus, and cerebellum [3,4]. AD is also accompanied by massive loss of mature functional neurons, synaptic dysfunction, dysregulation of neurotrophic factors, and neuroinflammation [5,6]. These neurodegenerative processes lead to brain atrophy and irreversible cognitive decline [7].

Despite extensive research since the early 20th century, the etiology and pathogenesis of this multifactorial disease are not fully understood, and effective treatment remains challenging [1,7]. Pharmacological agents commonly used to treat AD, such as donepezil, rivastigmine, galantamine, and memantine, help relieve symptoms and improve patients’ quality of life [7]. More recently developed anti-Aβ monoclonal antibodies such as lecanemab have shown efficacy in improving cognitive function in clinical trials [8]. These therapies can cause adverse effects, including headache, edema, and microhemorrhages [7,8], and while they may slow neurodegeneration, they do not promote regeneration of CNS tissue.

Transplantation of genetically modified cells expressing therapeutic transgenes is an emerging strategy for AD treatment. Neurotrophic factors are frequently used as therapeutic transgenes [9,10,11]. In neurodegenerative conditions, neurotrophin-3 (NT-3) is considered a key factor that not only supports neuronal survival but also stimulates axonal growth and synaptic transmission, which are critical for restoring lost neuronal connections in AD [11,12].

Exogenous delivery of NT-3 can be achieved by transplanting cells transduced with viral vectors encoding the NT-3 transgene into the affected area. Cells with inherent regenerative potential can be used as carriers for transgene delivery [13,14,15]. In particular, transplantation of olfactory ensheathing cells (OECs) derived from the olfactory mucosa has been widely applied in CNS disorders [16,17]. OECs are known to survive following transplantation, migrate within host tissue, secrete neurotrophic factors, promote axonal growth and remyelination, and phagocytose cellular debris in vivo [18,19], and improve cognitive outcomes, as shown in our previous studies [20,21].

However, despite the clear individual advantages of both OECs and NT-3, the therapeutic efficacy of transplanting NT-3-expressing transduced ensheathing cells from the olfactory mucosa in experimental AD models has not been investigated. We hypothesized that the transplantation of NT-3-overexpressing OECs would provide a superior dual-action therapeutic effect in AD by combining the cellular component with the target transgene.

Therefore, the aim of the current study was to evaluate the therapeutic potential of OECs transduced with an Ad5RGD-CAG-NT-3 adenoviral vector in a rat stereotaxic model of AD. We performed behavioral tests and biochemical measurements of Aβ, pTau, and NT-3 levels in brain tissue after transplantation of NT-3-transduced and non-transduced OECs. All OEC preparations improved cognitive performance, but only NT-3-transduced OECs reduced brain Aβ1–42 and increased NT-3 levels. The efficacy of NT-3-expressing OECs underscores the potential of such cell-based therapy as a novel treatment approach for AD.

## 2. Materials and Methods

### 2.1. Experimental Animals

Female Wistar rats, 4 months old (n = 75, body weights of 245 ± 15 g), were used in this study. Rats were obtained from the Pushchino Branch of IBCh RAS. (Pushchino, Russia) Animals were healthy, not previously used in experiments, and acclimatized for at least 7 days in an animal facility before the study.

The study was approved by the local Ethics Committee of the V. P. Serbsky National Medical Research Center for Psychiatry and Narcology (protocol code 01/25, date of approval 22 May 2025). All animal procedures conformed to Directive 2010/63/EU of the European Parliament and of Council on the protection of animals used for scientific purposes. Animals were housed at 21–24 °C with 50–70% relative humidity under a 12 h light/12 h dark cycle, with *ad libitum* access to food and water. Animals from different groups were housed under identical environmental conditions. All manipulations were performed according to the same protocol for all groups to minimize the potential confounding factors.

**Figure 1:**
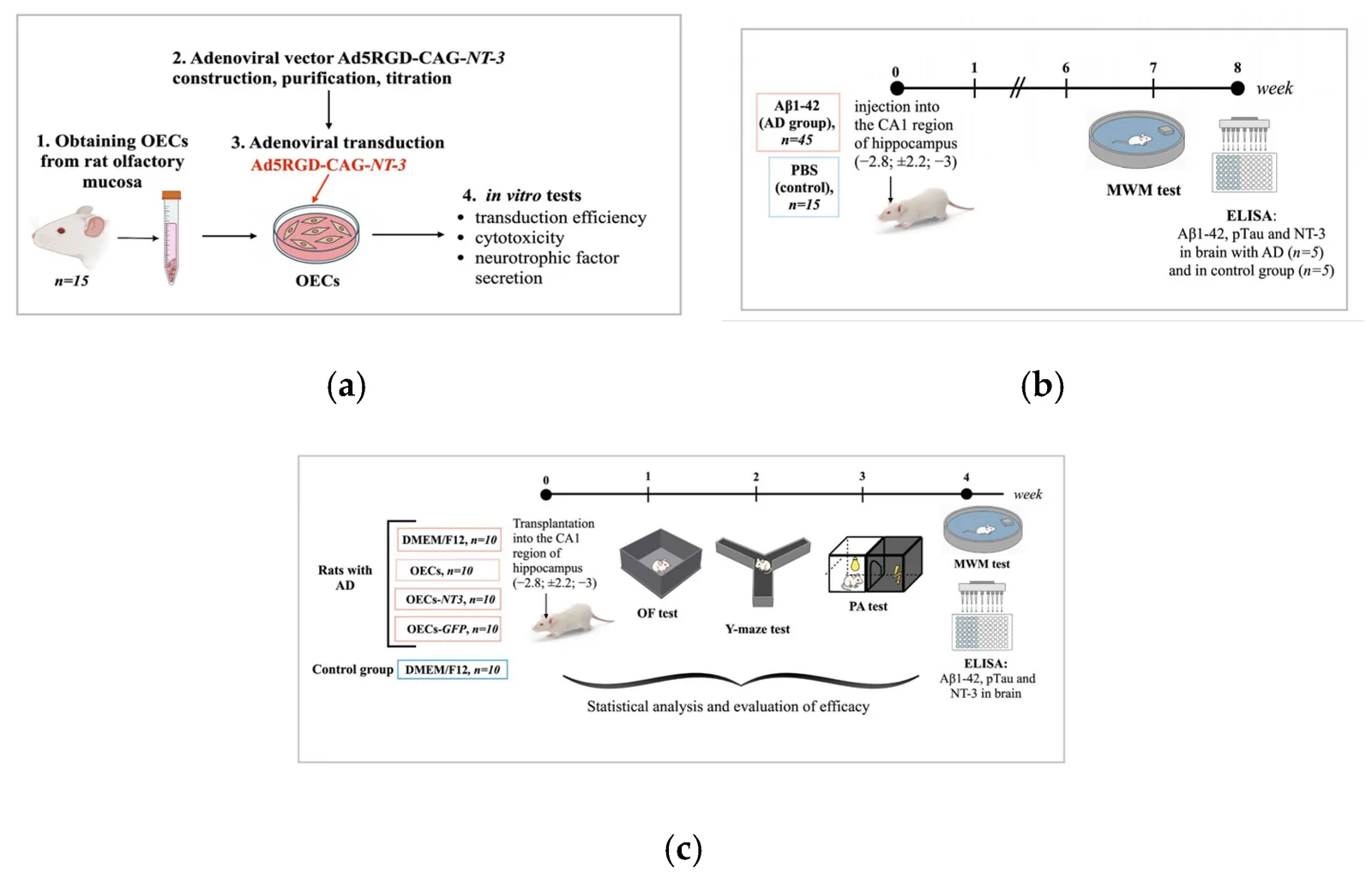
Experimental scheme. (**a**) Generation of Ad5RGD-CAG-NT-3-transduced OECs. (**b**) Modeling and validation of the experimental AD. (**c**) Assessment of the efficacy of Ad5RGD-CAG-NT-3-transduced OEC transplantation. OECs, olfactory ensheathing cells; AD, Alzheimer’s disease; MWM, Morris water maze; OF, open field; Y-maze, Y-maze; PA, passive avoidance test.

The experimental design is shown in Figure 1. The animal inclusion and exclusion criteria were determined *a priori*. Inclusion required a baseline healthy status, the absence of visible pathologies, and successful completion of the anhedonia test, whereas failure in the AD model validation (Section 2.5.1) was defined as an exclusion criterion for experimental animals. Animals that met the inclusion criteria were randomly allocated to treatment groups using simple randomization.

### 2.2. Isolation of OECs

Ensheathing cells from the olfactory mucosa (OECs) were isolated from the olfactory mucosa of female rats (n = 15) as previously described [22]. OECs were cultured in 100 mm Petri dishes (Corning, Durham, NC, USA) at 37 °C in 5% CO_2_ (Binder, Tuttlingen, Germany) in complete culture medium consisting of DMEM/F12 (Gibco, Grand Island, NY, USA), 10% FBS (HyClone, Logan, UT, USA), 2 mM L-glutamine (Gibco, USA), 2 mM streptomycin + penicillin (Gibco, USA), and 60 μg/mL gentamicin (Gibco, USA) until the monolayers reached ~80–90% confluence. Cells were passaged using 0.1% trypsin (Gibco, USA) and plated at a density of 1 × 10^4^ cells per cm^2^. OECs from passages 3 and 4 were used in the experiments.

### 2.3. Design of Adenoviral Vectors

Adenoviral vectors encoding NT-3 (Ad5RGD-CAG-NT-3; deltaE1A) and enhanced green fluorescent protein (EGFP) (Ad5RGD-CAG-EGFP; deltaE1A) were developed on the basis of the Department of Applied and Fundamental Neurobiology of the V. P. Serbsky National Medical Research Center for Psychiatry and Narcology. These vectors were constructed based on the Ad5-delta-24-RGD vector obtained earlier [23], transgene sequences encoding NT-3 proteins (Sino Biologicals, Beijing, China) and EGFP (from the Collection of the Department) were inserted (Table 1). Construction and validation of recombinant adenoviral plasmids are shown in Figure S1. The design of adenovectors containing transgenes, their production, purification, storage, and titration were carried out in accordance with the protocols described earlier [23]. The oligonucleotide sequences used for cloning, recombination, and sequencing are listed in Table 1. All oligonucleotides were ordered from Evrogen (Moscow, Russia) or Syntol (Moscow, Russia).

### 2.4. Adenoviral Vectors Transduction

OECs were transduced in vitro with Ad5RGD-CAG-NT-3 and Ad5RGD-CAG-EGFP to evaluate transduction efficiency, cytotoxicity, and NT-3 secretion. The human adenocarcinoma cell line A549 was used as a control. Experiments were performed in three independent replicates.

#### 2.4.1. Transduction Efficiency

The control vector Ad5RGD-CAG-EGFP (infectious titer: 3.7 × 10^10^ IFU/mL) was used to assess transduction efficiency. Serial dilutions of the viral preparations were prepared (200, 66, 22, 7.4, 2.5, 0.8 IFU). Cells were placed in 96-well plates (Corning, USA) at a density of 5 × 10^4^ cells per well in 80 μL of complete medium, and 20 μL of viral inoculum was added to each well. After 48 h, EGFP expression was assessed using fluorescence microscopy (Nikon Eclipse Ti2-FL, Tokyo, Japan). Transduction efficiency was determined as the proportion of EGFP-positive cells relative to the total cells observed under phase contrast. Transduction was considered effective if more than 80% of cells expressed EGFP [24].

#### 2.4.2. Cytotoxicity

To evaluate Ad5RGD-CAG-NT-3 cytotoxicity, viral dilutions were prepared (100, 33, 11, 3.7, 1.2, 0.4 IFU). Cells were plated in 96-well plates at 5 × 10^4^ cells per well in 80 μL of complete medium, and 20 μL of viral inoculum was added. On the following day, wells were supplemented with 100 μL of complete medium. After 6–7 days, 100 μL of resazurin dye (Biotium, Fremont, CA, USA) was added to each well following medium aspiration. After 4 h of incubation, resorufin fluorescence (excitation 560 nm, emission 590 nm) was measured using a CLARIOstar^®^ Plus multimode plate reader (BMG LABTECH, Ortenberg, Germany). Adenoviral vector cytotoxicity was evaluated by cell viability relative to non-transduced cells; a viability of ≥80% was considered acceptable [25].

#### 2.4.3. In Vitro NT-3 Secretion

To quantify NT-3 secretion in vitro following transduction, cells were plated in 24-well plates (Corning, USA) at 1 × 10^6^ cells per well in 250 μL and infected in suspension with Ad5RGD-CAG-NT-3 at MOIs of 30 and 10, respectively. Conditioned medium was collected 48 h post-infection, cellular debris was pelleted at 1000 g (Mini-Spin Eppendorf, Leipzig, Germany), and NT-3 levels were determined by enzyme-linked immunosorbent assay (ELISA) according to the manufacturer’s instructions (FineTest, Wuhan, China). Optical density was measured on a 2300 Multilabel Reader EnSpire (PerkinElmer, Waltham, MA, USA) at 450 nm.

### 2.5. Experimental Model of Alzheimer’s Disease

AD was modeled by the intrahippocampal administration of Aβ1–42 (1 mg/mL) (n = 45) (ElabScience, Wuhan, China) [26]. Control animals received bilateral injections of PBS (Servicebio, China) (n = 15). Animals were anesthetized with vesotil (40 mg/kg) (Vesstem, Moscow, Russia) and xylazine (5 mg/kg) (Alfasan International B.V., Woerden, The Netherlands), and stereotaxic access to the skull was obtained for visualization of the bregma and lambda. Rats were fixed in a stereotaxic frame (RWD, Shenzhen, China), and Aβ1–42 was infused bilaterally into the CA1 region (x = −2.8, y = ±2.2, z = −3) of the hippocampus at a rate of 1 μL/min according to the Paxinos and Watson rat brain atlas [27]. Intact and sham control groups were not included in this study because our previous work revealed no differences between these groups in this AD model [21]. However, these groups were included in the Supplementary Materials to further confirm the absence of significant differences in behavioral and biochemical parameters among the control groups (Figure S2).

#### 2.5.1. Morris Water Maze (MWM)

To validate the model, cognitive testing in the Morris water maze was performed 7 weeks after Aβ1–42 administration [28]. The MWM is the most informative and adequate test for assessing spatial memory changes in rodents during neurodegeneration [29]. The MWM consisted of a cylindrical pool (150 cm diameter, 40 cm height) filled to a depth yielding a water temperature of 22–23 °C, containing a hidden platform. Training included eight swims from different start points (seven distinct start locations in total), each lasting up to 60 s; if an animal failed to find the platform, it was placed on it for 15 s. After 48 h, a probe test for locating the hidden platform was performed. Animals with AD that required more than 40 s to locate the platform were selected for subsequent therapeutic experiments [30,31].

#### 2.5.2. ELISA

To assess Aβ1–42, pTau, and NT-3 levels in the brain, ELISA was performed 8 weeks after AD modeling (controls, n = 5; experimental, n = 5; samples selected by randomization) (Table 1). The hippocampus, part of the corpus callosum, and a cortical region surrounding the stereotaxic injection site were harvested. Samples were homogenized using a SilentCrusher (Heidolph, Schwabach, Germany) in cold PBS (Servicebio, Wuhan, China) containing a protease inhibitor cocktail mini-tablet (EDTA-free) (MCE, Monmouth Junction, NJ, USA) at a ratio of 1 g tissue per 5 mL buffer. Homogenates underwent three freeze–thaw cycles and were sonicated using an Elmasonic S 300H (Elma, Singen, Germany). Supernatants were collected after centrifugation at 16,000 rpm for 15 min (Mini-Spin Eppendorf, Germany). ELISAs were performed according to the manufacturer’s instructions (FineTest, China). Optical density was measured using a 2300 Multilabel Reader EnSpire (PerkinElmer, Waltham, MA, USA) at 450 nm.

### 2.6. Transplantation of Ad5RGD-CAG-NT-3-Transduced OECs

OECs were transduced with Ad5RGD-CAG-NT-3 and Ad5RGD-CAG-EGFP in a minimal volume of complete culture medium while maintaining an MOI of 30 for 2 h. After transduction, the required volume of complete medium was added for continued culture.

Non-transduced OECs were cultured for comparison.

Two days after transduction, cell suspensions were prepared for transplantation (Table 2). Each sample contained 0.75 × 10^6^ cells in 10 μL of DMEM/F12 (Gibco, USA).

Prior to transplantation, the animals were anesthetized and fixed in a stereotaxic frame, as described in the “Experimental model of Alzheimer’s disease” section. Cell preparations were injected into the CA1 region of both hippocampi (x = −2.8, y = ±2.2, z = −3) at a rate of 1 μL/min. Postoperatively, animals received ketoprofen at 5 mg/kg (VIK—Animal Health, Belgorod, Russia).

The animals were then randomly divided into equal groups:

1. Control (PBS + DMEM/F12, n = 10);
2. Aβ1–42:
  - DMEM/F12 (Aβ1–42 + DMEM/F12, n = 10),
  - OECs (Aβ1–42 + OECs, n = 10),
  - Ad5RGD-CAG-NT-3-transduced OECs (Aβ1–42 + OECs-NT-3, n = 10),
  - Ad5RGD-CAG-EGFP-transduced OECs (Aβ1–42 + OECs-EGFP, n = 10).

The following experimental groups were formed to evaluate the efficacy of cell transplantation for 4 weeks (Table 2 and Figure 1).

### 2.7. Behavioral Assessment

All behavioral procedures were conducted between 9:00 am and 3:00 pm in a quiet, dimly lit room. Rats were brought to the testing room 30 min before each test for habituation. Apparatuses were cleaned with 75% ethanol between trials to eliminate olfactory cues. Behavioral assessments were conducted for up to 4 weeks to assess the therapeutic efficacy of transplanted OECs in experimental AD. We compared behavioral outcomes between the experimental and control groups following cell transplantation over a 4-week period (Figure 1).

#### 2.7.1. Open Field Test

In week 1, the open field test was performed to evaluate mean velocity, distance traveled, freezing, and time spent in the center. This test assesses locomotor and exploratory activity as well as anxiety-like behavior. The apparatus consisted of a white plastic square arena (120 × 120 × 50 cm) illuminated at ~25 lux. Each animal was placed in the upper-right corner and recorded for 5 min using an overhead camera. Parameters were tracked using AnyMaze software version 2.0 (Stoelting Co., Wood Dale, IL, USA).

#### 2.7.2. Y-Maze

In week 2, the Y-maze test was used to assess short-term working memory and spatial memory. The Y-maze consisted of three identical numbered arms (40 × 19 × 18 cm). Animals were placed in the center and allowed to explore freely for 8 min. The sequence and number of arm entries were recorded. The number of spontaneous alternation triplets (three consecutive, non-repeating arm entries) was counted and used to calculate the spontaneous alternation index.

#### 2.7.3. Passive Avoidance

In week 3, a passive avoidance test was performed to evaluate learning and memory. The test was conducted in a “Shelter” apparatus (Neurobiotics, Zelenograd, Russia), a plastic chamber (40 × 30 × 36 cm) with a grid floor separated by a partition with an 8 × 8 cm opening into light and dark compartments. During training, rats were placed in the light compartment and, upon full entry into the dark compartment with all four paws, received a foot shock of 0.1 mA for 1 s to return them to the light compartment. Testing was conducted on the third day after training. Rats were placed again in the light compartment and observed for 300 s. The time spent in the light compartment was recorded as passive avoidance.

#### 2.7.4. MWM Retest

In week 4, the MWM was retested to assess long-term memory retention. The animals’ ability to locate the hidden platform (placed at the same location as before transplantation one month earlier) was evaluated; a maximum of 60 s was allowed to locate the platform.

#### 2.7.5. Modified MWM

On the same day, animals underwent retraining in a modified MWM to assess spatial reorientation. The modification consisted of relocating the hidden platform. Training included four trials, starting from three different start points. Each trial lasted up to 60 s. Final testing was performed 48 h later, and the latency to reach the hidden platform from the designated start point was recorded.

### 2.8. Biochemical Assessment

Aβ1–42, pTau, and NT-3 levels in rat brain samples were quantified using ELISA 4 weeks after transplantation. The samples were prepared as described in Section 2.5. ELISAs were performed according to the kit manufacturers’ protocol (FineTest, China).

### 2.9. Statistical Analysis

Behavioral testing and biochemical analyses were performed by investigators blinded to treatment allocation. No animals or data points were excluded during the experiment or analysis. Statistical analyses and graphing were performed using GraphPad Prism version v10.3.1 (GraphPad Software, San Diego, CA, USA). The normality of the data within each group was assessed using the Shapiro–Wilk test. Depending on the data distribution, quantitative variables were presented as mean ± standard error of the mean (M ± SEM) or median and interquartile range (Me ± IQR).

For comparisons between two independent groups, Student’s *t*-test or the Mann–Whitney U test was used as appropriate. For multiple comparisons among three or more groups, researchers employed one-way ANOVA with Tukey’s post hoc test or Holm–Sidak test for parametric data, and the Kruskal–Wallis test with Dunn’s post hoc test for nonparametric data. A *p*-value of <0.05 was considered statistically significant.

## 3. Results

### 3.1. Transduction with Ad5RGD-CAG-EGFP Induces EGFP Expression In Vitro

**Figure 2:**
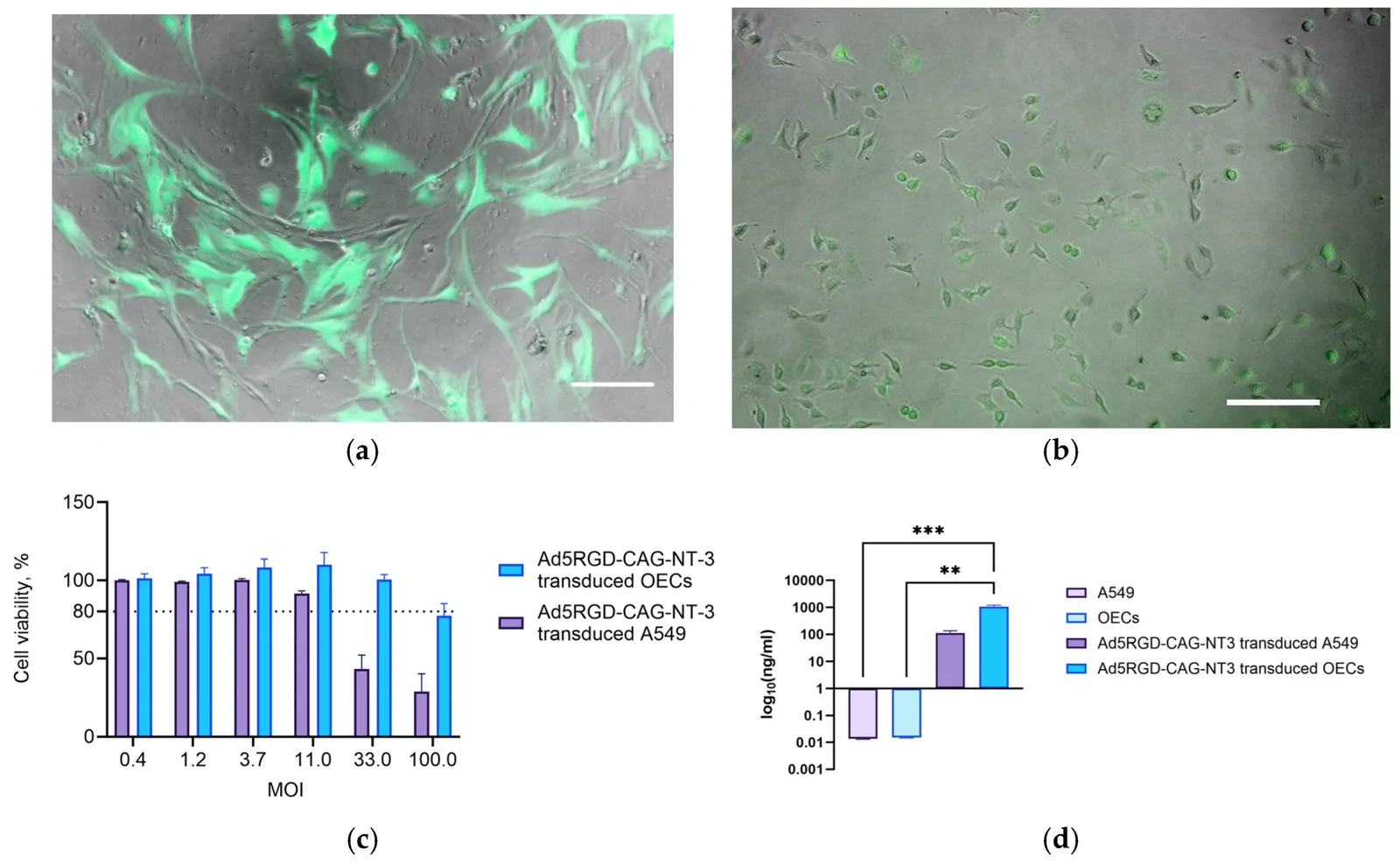
Transduction efficiency of Ad5RGD-CAG-EGFP and cytotoxicity of Ad5RGD-CAG-NT-3 in vitro. (**a**,**b**) Merged phase-contrast and fluorescence microscopy images (excitation at 488 nm) of (**a**) OECs transduced at MOI = 22 and (**b**) A549 cells transduced at MOI = 7.4, demonstrating a transduction efficiency exceeding 80%. Scale bars = 50 µm. (**c**) Cytotoxicity profiles of Ad5RGD-CAG-NT-3 in OECs and A549 cells evaluated one week post-transduction. Data are presented as mean ± SEM. Group differences were assessed using a two-way ANOVA followed by Tukey’s multiple-comparisons test. (**d**) NT-3 secretion by rat OECs and A549 cells 72 h after Ad5RGD-CAG-NT-3 transduction. Data are presented as log_10_-transformed protein concentrations (mean ± SEM). Group differences were assessed using Kruskal–Wallis with Dunn’s post hoc test. Significance: ** *p* < 0.0061, *** *p* < 0.0003.

To determine the optimal multiplicity of infection (MOI) for subsequent experiments, we accessed the transduction efficiency of the Ad5RGD-CAG-EGFP. In the A549 cell line, transduction efficiency plateaued at MOI ≥ 2.8 and exceeded 80% (89.2 ± 5.4) (Figure 2b—MOI 7.4). For OECs, transduction efficiency reached >80% (86.5 ± 2.1) at MOI ≥ 22, whereas at MOI ≤ 7.4, the proportion of transduced cells was below 80% (Figure 2a—MOI 22). Thus, an MOI of 22 or higher is sufficient for efficient transduction of rat OECs with the Ad5RGD vector driven by the CAG promoter. Transduction efficiency at the other tested MOIs is shown in Figure S3.

### 3.2. Cytotoxicity of the Adenoviral Vector

Cells were transduced with Ad5 vectors at various MOIs to evaluate Ad5RGD-CAG-NT-3 cytotoxicity. We performed a resazurin (AlamarBlue) cytotoxicity assay and observed a pronounced cytotoxic effect in A549 cells at MOI ≥ 33 on day 6 post-transduction (Figure 2c). In contrast, OECs demonstrated 100% viability after transduction across the MOI range tested (0.4–33 IFU) (Figure 2c). Therefore, any MOI within the range 0.4–33 may be used for subsequent in vivo transplantation experiments with Ad5RGD-CAG-NT-3-transduced OECs.

In this study, an MOI of 30 was employed. At this MOI, Ad5RGD-CAG-EGFP transduction efficiency exceeded 80%, and no marked cytotoxicity of Ad5RGD-CAG-NT-3 was detected in transduced OECs.

### 3.3. Transduced OECs Secrete NT-3 In Vitro

To confirm successful transduction, NT-3 secretion was measured in the conditioned media 3 days after transduction using ELISA (Figure 2d). Non-transduced OECs and A549 cells secreted only basal levels of NT-3, with comparable concentrations (0.015 ± 0.0009 ng/mL and 0.013 ± 0.002 ng/mL, respectively) (Table 3). In contrast, Ad5RGD-CAG-NT-3-transduced OECs secreted 1064.398 ± 324.595 ng/mL NT-3, representing an approximately 71,000-fold increase compared with non-transduced OECs (*p* = 0.0061). Similarly, transduced A549 cells secreted 120,531 ± 38.435 ng/mL NT-3, whereas non-transduced A549 cells produced only basal levels (0.013 ± 0.002 ng/mL). Thus, genetic modification of OECs via transduction with the Ad5RGD-CAG-NT-3 adenoviral vector demonstrated high NT-3 levels in vitro.

### 3.4. Validation of the Experimental AD Model

The experimental AD model was validated by evaluating spatial memory and biochemical alterations in the brain after Aβ1–42 injection.

#### 3.4.1. Intrahippocampal Injection of Aβ1–42 Impairs Spatial Orientation

**Figure 3:**
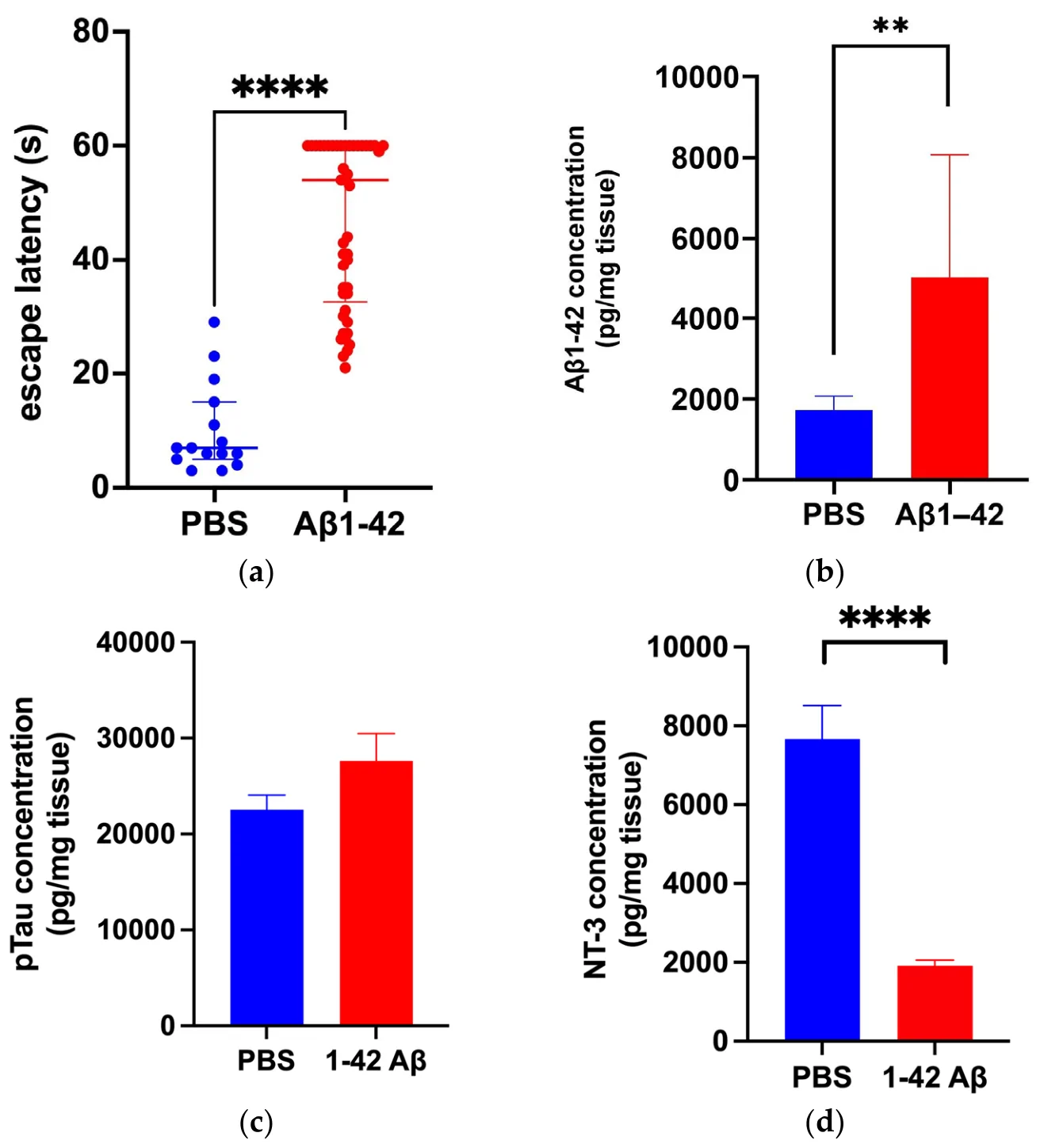
(**a**) Latency to locate the hidden platform 48 h after training in the Morris water maze for PBS controls and Aβ1–42-treated animals. Data are presented as median ± IQR; *p* < 0.0001, Mann–Whitney U test. (**b**–**d**) Comparative quantification of (**b**) Aβ1–42, (**c**) pTau and (**d**) NT-3 in brain tissue from Aβ1–42 and PBS groups at 8 weeks via ELISA. (**b**) Data are presented as median ± IQR; Mann–Whitney U test. (**c**,**d**) Data are presented as mean ± SEM; Student’s *t*-test. Significance: ** *p* < 0.0021, **** *p* < 0.0001.

To assess the functional implication of Aβ1–42 administration, we evaluated the cognitive performance in the Morris water maze. Seven weeks after modeling, animals that received Aβ1–42 required a mean latency of 47 s to locate the hidden platform (Figure 3a), whereas PBS-treated control animals required only 13 s (*p* < 0.0001). The prolonged latency in Aβ1–42-treated animals in the Morris test indicates impaired spatial orientation and confirms the validity of our experimental AD model [28].

#### 3.4.2. Brain Aβ1–42, pTau, and NT-3 Levels 8 Weeks After Aβ1–42 Injection

To confirm that observed effects were due to increased Aβ1–42 levels, we studied the brain specimens from animals with experimental AD. Aβ1–42 levels were significantly increased compared to controls (*p* = 0.0014) at 8 weeks post-injection (Figure 3b), while the levels of pTau tended to increase; this trend remained insignificant (*p* = 0.117) (Figure 3c). Also Aβ1–42 administration produced a significant fourfold reduction in NT-3 levels in brain tissue compared to PBS-injected controls (*p* < 0.0001) (Figure 3d).

To conclude eight weeks post-injection, Aβ1–42 administration significantly increases brain Aβ1–42 levels and causes a fourfold reduction in NT-3, with no significant decrease in pTau level.

### 3.5. Transplantation of Ad5RGD-CAG-NT-3-Transduced OECs Improves Cognitive Function in Aβ1–42-Treated Rats

Open field test

**Figure 4:**
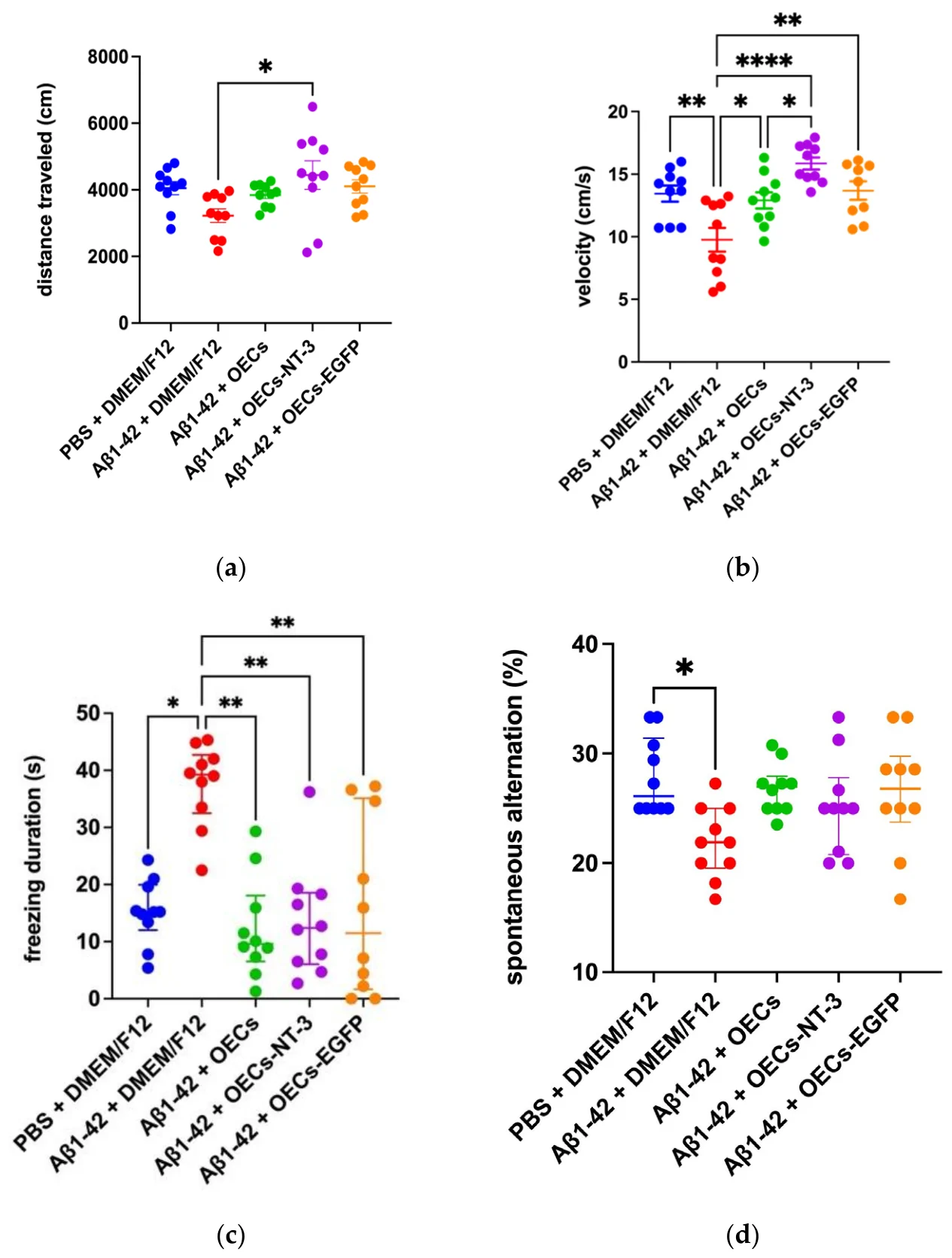

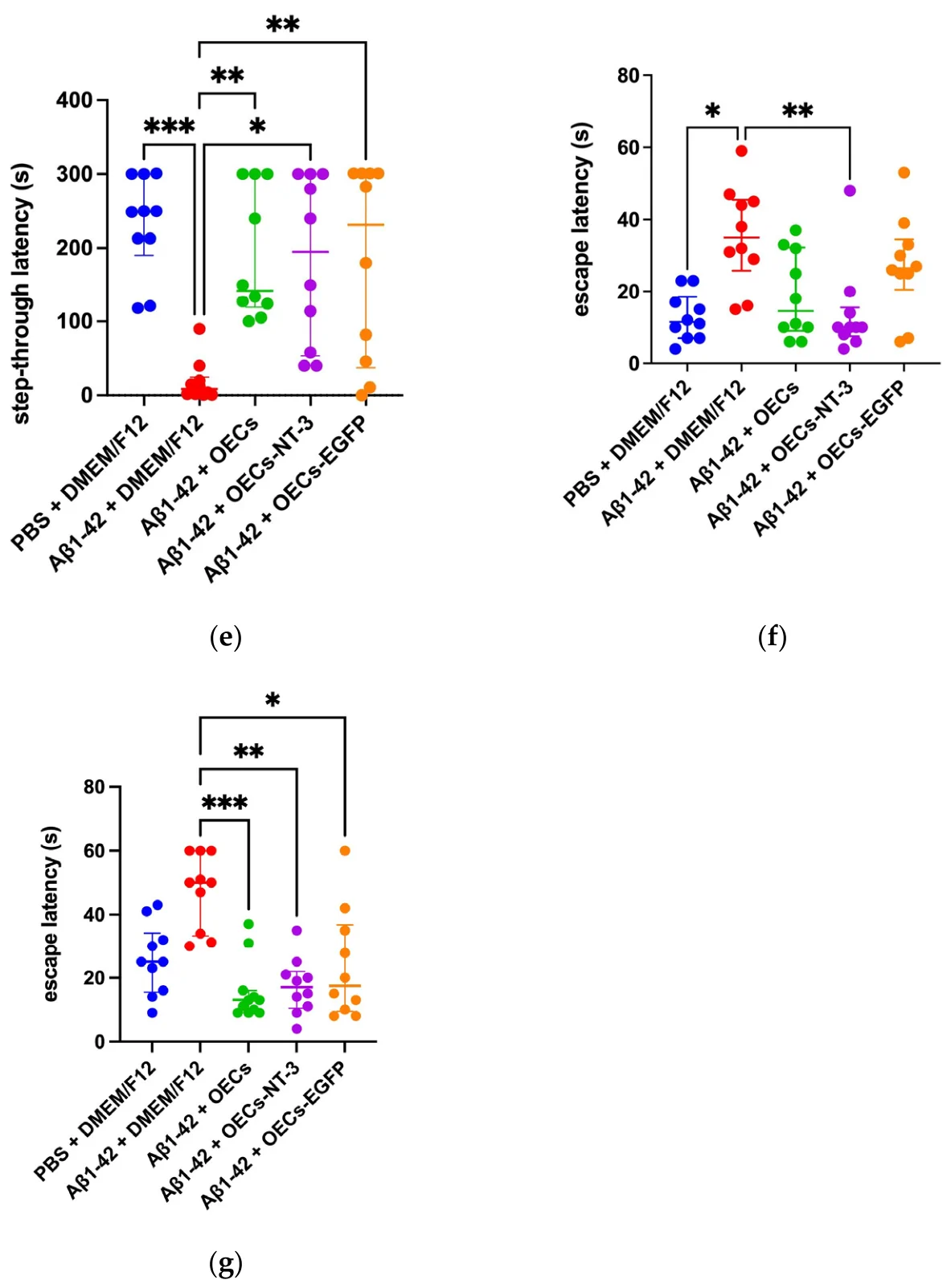
Behavioral testing results after transplantation of various rat OEC-based preparations: (**a**) distance traveled, (**b**) movement speed, and (**c**) freezing duration in the Open Field test (week 1); (**d**) Y-maze (week 2); (**e**) passive avoidance (week 3); (**f**,**g**) Morris water maze at week 4: (**f**) retest and (**g**) modified test. Data are presented (**a**,**b**) as mean ± SEM and (**c**–**g**) as median ± IQR. Group comparisons: one-way ANOVA with Tukey’s post hoc test (**a**–**c**) and Kruskal–Wallis with Dunn’s post hoc test (**d**–**g**). Significance: * *p* < 0.0332, ** *p* < 0.0021, *** *p* < 0.0002, **** *p* < 0.0001.

One week after transplantation, the total distance traveled was significantly greater in the Aβ1–42 + OECs–NT-3 group (*p* = 0.0106) (Figure 4a). No significant differences were observed among the Aβ1–42 + OECs and Aβ1–42 + OECs–EGFP groups compared to untreated Aβ1–42 animals. No differences relative to the PBS control group were detected.

Mean movement speed was significantly higher in the Aβ1–42 + OECs (*p* = 0.0223), Aβ1–42 + OECs–NT-3 (*p* < 0.0001), and Aβ1–42 + OECs–EGFP (*p* = 0.0033) groups than in the Aβ1–42 + DMEM/F12 group (Figure 4b). Moreover, the Aβ1–42 + OECs–NT-3 group exhibited a significantly higher mean movement speed than the Aβ1–42 + OECs group (*p* = 0.0368). The Aβ1–42 + DMEM/F12 group also showed a significantly higher mean movement speed than the control group (*p* = 0.005).

The freezing duration was significantly longer in untreated Aβ1–42 animals than in PBS controls (*p* < 0.0001). Treatment with OECs, OECs–NT-3, or OECs–EGFP significantly reduced freezing duration compared to the Aβ1–42 + DMEM/F12 group (all *p* < 0.0001). In addition, freezing duration was significantly longer in the Aβ1–42 + DMEM/F12 group than in the control group (*p* = 0.0001).

The time spent in the central area was significantly increased in the Aβ1–42 + OECs–NT-3 group compared to that in the untreated Aβ1–42 group (*p* = 0.0339) (Figure S5). Untreated Aβ1–42 animals spent significantly less time in the central area than the PBS control group (*p* = 0.007). No significant differences were observed between the Aβ1–42 + OECs or Aβ1–42 + OECs–EGFP groups and the untreated Aβ1–42 group.

Y-maze

Two weeks after transplantation, the untreated Aβ1–42 + DMEM/F12 group showed a trend toward reduced spontaneous alternation compared to all treatment groups, although these differences were not statistically significant (Figure 4d). In contrast, spontaneous alternation was significantly lower in the untreated Aβ1–42 + DMEM/F12 group than in the control group (*p* = 0.0201).

Passive avoidance

Three weeks after transplantation, passive avoidance latency was significantly lower in the Aβ1–42 + DMEM/F12 group compared to Aβ1–42 + OECs (*p* = 0.0092), Aβ1–42 + OECs + NT-3 (*p* = 0.0194) and Aβ1–42 + OECs–EGFP (*p* = 0.0076) groups (Figure 4e). In addition, the Aβ1–42 + DMEM/F12 group showed significantly shorter latency compared to the PBS + DMEM/F12 control group (*p* = 0.0007).

Morris water maze (MWM)

Four weeks after transplantation, animals in the Aβ1–42 + OECs–NT-3 group showed a significantly reduced latency to locate the hidden platform compared with the untreated Aβ1–42 + DMEM/F12 animals (*p* = 0.01) (Figure 4f). In contrast, untreated Aβ1–42 animals required significantly longer to locate the hidden platform than the PBS + DMEM/F12 controls (*p* = 0.0145).

In the modified MWM, significant reductions in platform-finding latency compared with the Aβ1–42 + DMEM/F12 group were observed in the Aβ1–42 + OECs (*p* = 0.0003), Aβ1–42 + OECs + NT-3 (*p* = 0.0032) and Aβ1–42 + OECs–EGFP (*p* = 0.0241) groups (Figure 4g).

These findings indicate that OEC transplantation, including Ad5RGD-CAG-NT-3-transduced OECs, modulates behavioral performance in Aβ1–42-treated rats, with early improvements in behavioral responses during the first week after transplantation and subsequent improvements in memory-related tasks, including passive avoidance and spatial learning, as assessed by the MWM.

### 3.6. Effect of Ad5RGD-CAG-NT-3-Transduced OEC Transplantation on Brain Aβ1–42 and pTau Levels

**Figure 5:**
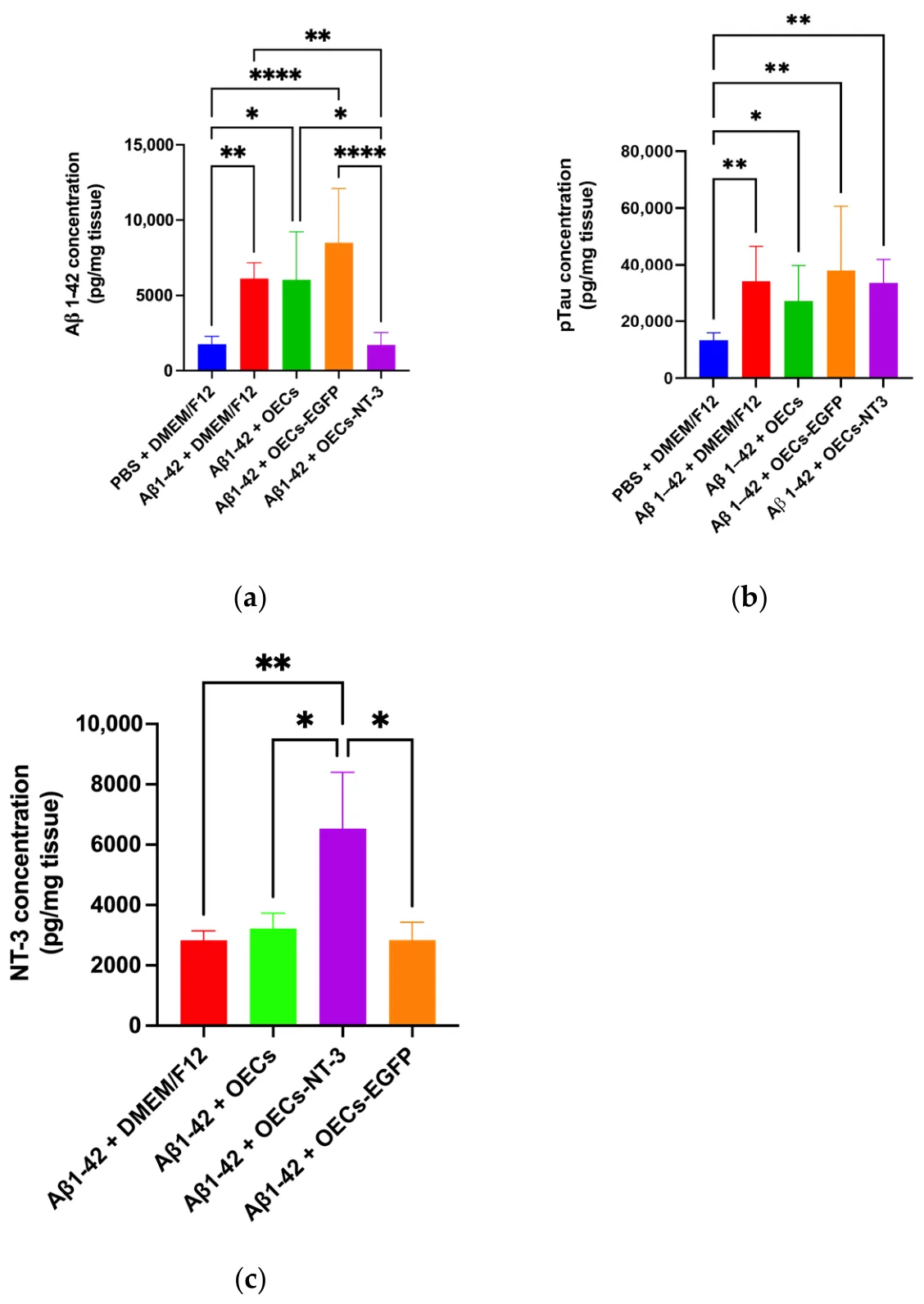
Brain tissue levels of (**a**) Aβ1–42, (**b**) pTau, and (**c**) NT-3 in control and experimental AD groups after transplantation of various rat OEC-based preparations. (**a**,**b**) Data as median ± IQR; (**c**) data as mean ± SEM. Group comparisons: Kruskal–Wallis with Dunn’s post hoc test (**a**,**b**) and one-way ANOVA with Holm–Sidak post hoc test (**c**). Significance: * *p* < 0.0332, ** *p* < 0.0021, **** *p* < 0.0001.

Four weeks after transplantation, animals in the Aβ1–42 + OECs–NT-3 group exhibited significantly lower brain Aβ1–42 levels compared to untreated Aβ1–42 + DMEM/F12 animals (*p* = 0.0041) (Figure 5a). Aβ1–42 levels in the Aβ1–42 + OECs–NT-3 group were also significantly lower than those in Aβ1–42 + OECs (*p* = 0.0102) and Aβ1–42 + OECs–EGFP (*p* < 0.0001) groups (Figure 5a). Notably, Aβ1–42 amounts in the Aβ1–42 + OECs–NT-3 group were comparable to those in the PBS + DMEM/F12 control group.

In contrast, Aβ1–42 levels were significantly elevated relative to PBS controls in the following groups: Aβ1–42 + DMEM/F12 (*p* = 0.0049), Aβ1–42 + OECs (*p* = 0.0124) and Aβ1–42 + OECs–EGFP (*p* < 0.0001) (Figure 5a).

Four weeks after transplantation, pTau levels were significantly higher in all Aβ1–42 groups compared with PBS + DMEM/F12 controls: Aβ1–42 + DMEM/F12 (*p* = 0.0012), Aβ1–42 + OECs (*p* = 0.0343), Aβ1–42 + OECs–EGFP (*p* = 0.0024) and Aβ1–42 + OECs–NT-3 (*p* = 0.0034) (Figure 5b). No significant reduction in pTau levels was observed following transplantation of all OECs grafts.

These results showed that Ad5RGD-CAG-NT-3-transduced OEC transplantation significantly reduced brain Aβ1–42 levels to control values after 4 weeks without affecting pTau accumulation.

### 3.7. Transplanted Transduced OECs Increase NT-3 Content in the Brain of Animals with AD Model

NT-3 brain levels were significantly higher in the Aβ1–42 + OECs–NT-3 group than in untreated Aβ1–42 + DMEM/F12 animals (*p* = 0.0094) and higher than in other cell-treated groups (Aβ1–42 + OECs, *p* = 0.0283; Aβ1–42 + OECs–EGFP, *p* = 0.025) four weeks after transplantation (Figure 5c).

No significant changes in brain NT-3 content were observed in the Aβ1–42 + OECs and Aβ1–42 + OECs–EGFP groups compared with untreated Aβ1–42 + DMEM/F12 animals.

Importantly, transplanted NT-3-transduced OECs significantly increased brain NT-3 levels 4 weeks post-transplantation.

## 4. Discussion

The efficacy of cell-based gene therapy depends on the appropriate selection of both the cellular carrier and therapeutic transgene. For neurodegenerative disorders such as AD, combining cells with intrinsic regenerative properties and targeted neuroprotective factors represents a promising strategy to promote neuronal recovery and modulate disease progression.

In this study, NT-3-expressing OECs were generated using an adenoviral vector based on human adenovirus serotype 5 (Ad5). The use of Ad5 vectors is supported by their favorable safety profile, including the absence of genomic integration, high transgene capacity, and efficient transgene expression in vivo [32,33]. Because rodent cells are less susceptible to Ad5 infection than human cells [34], optimization of in vitro transduction conditions was required to achieve efficient gene delivery into rat OECs. Under the optimized conditions, the Ad5RGD-CAG-NT-3 vector efficiently transduced rat OECs without inducing cytotoxicity and increased NT-3 secretion by approximately 71,000-fold compared to the near-undetectable basal levels observed in non-transduced cells. This finding is consistent with previous reports demonstrating that OECs produce low or undetectable basal endogenous levels of NT-3 [35,36] and provides a rationale for the development of genetically engineered NT-3-expressing cell products for the treatment of spinal cord injury, traumatic brain injury, and other neurological disorders [18,36]. In our study, transduced rat OECs at passages 3–4 exhibited robust *de novo* NT-3 secretion, confirming the effective adenovirus-mediated transgene expression. To our knowledge, this is the first study to evaluate the therapeutic efficacy of NT-3-expressing OECs generated using the Ad5RGD-CAG-NT-3 vector in an AD experimental model.

The AD model was induced by stereotaxic injection of Aβ1–42 into the hippocampus [28], which is a brain region critically involved in memory consolidation [37,38]. This approach is among the most widely used experimental models for evaluating therapeutic interventions in AD research [26,39,40]. Our model successfully reproduces the key clinical features of AD, namely cognitive impairment and increased Aβ1–42 levels in the brain, which is consistent with the literature [41,42]. However, we detected no significant changes in pTau protein levels eight weeks after model induction, with only a minor upward trend observed. This finding is consistent with the temporal dynamics of acute Aβ1–42 injection models, where cognitive deficits may precede detectable tau-related changes [43], while more pronounced tau pathology generally requires longer disease progression or transgenic backgrounds. Therefore, behavioral impairments observed in the present model are likely associated primarily with early Aβ-induced synaptic dysfunction. We assessed concentrations of Aβ1–42 and pTau in the brain specimens but did not evaluate amyloid plaques and neurofibrillary tangle morphology, as stereotaxic administration of exogenous Aβ peptides does not fully reproduce the pathological structures observed in human AD. Such features are more commonly observed in transgenic AD models [44,45].

Our findings of reduced NT-3 levels in the brains of AD rats are important in view of the contradictory data already reported in previous studies. Variable NT-3 dynamics have been demonstrated both in the brains of patients [46] and in experimental AD models [47]. The NT-3 levels might be region-specific and influenced by disease progression, as well as by age-related changes in the brain during normal aging [48]. In our study, the decrease in NT-3 may be attributed to the choice of the investigated region (the hippocampus and surrounding area) as well as to the specific AD modeling protocol.

The Aβ1–42-induced experimental AD model allowed the evaluation of the therapeutic potential of NT-3-expressing OECs. Both NT-3-expressing and non-transduced OECs improved behavioral performance, highlighting the contribution of the OEC cellular component to functional recovery. This effect may be attributed to the intrinsic regenerative properties of OECs, including their high survival after transplantation, migratory capacity within the CNS, neuroprotective activity, ability to promote axonal regeneration and remyelination, phagocytose cellular debris, and modulate neuroinflammatory responses [17,49,50]. Restoration of neural connectivity through these mechanisms may contribute to improved neuronal signaling and, consequently, cognitive function [51].

Clinical studies of autologous OECs transplantation in patients with spinal cord injury [17] and AD [16] have demonstrated the feasibility and safety of this approach, supporting its potential for further therapeutic development. In our previous studies, we demonstrated that transplanted OECs survive and migrate within the brain over an 4-week observation period [20], as illustrated in Figure S4, and contribute to improved cognitive outcomes in rats with experimental AD [21]. Therefore, a cell-based gene therapy strategy combining OECs transplantation with NT-3 exogenous delivery represents an attractive approach for neurodegenerative disorders by modulation of the local microenvironment.

NT-3 is a promising candidate due to its role in neuroregeneration and neuronal survival, with reduced levels reported in AD patients [46,47] and linked to hippocampal neuronal loss, synaptic decline, and cognitive impairment [11,41]. We showed that only NT-3-expressing OECs reduced brain Aβ1–42 levels and increased NT-3 levels after transplantation suggesting that the behavioral benefits are primarily mediated by the intrinsic therapeutic properties of OECs, while Aβ1–42 reduction is associated with NT-3 overexpression. Similar neuroprotective effects have been reported for BDNF, which promotes neuronal survival and functional recovery and may also reduce cerebral amyloid burden [52,53,54]. We suggest that NT-3 overexpression has autocrine effects that increase their phagocytic capacity and secretory activity [55,56]. In turn, elevated NT-3 may promote Aβ1–42 clearance—rather than suppressing amyloid production—by enhancing microglial/astrocytic phagocytosis, shifting glial cells toward a neuroprotective phenotype, boosting enzymatic Aβ degradation, and facilitating glymphatic clearance [57,58]. These mechanisms remain hypothetical in the context of the present study and require further experimental validation.

Our efficacy findings of NT-3-expressing transduced cells are consistent with previous observations reported by Yan et al. In their study, bone marrow-derived mesenchymal stem cells genetically modified to express NT-3 were transplanted into rats with Aβ1–42-induced AD [11]. This treatment improved cognitive function through activation of the Wnt/β-catenin signaling pathway, thereby promoting neuronal survival, enhancing neurogenesis, and protecting synapses from Aβ-induced neurotoxicity [11].

Several other neurotrophic factors have also been investigated as therapeutic transgenes for cell-based gene therapy in AD. Wu et al. demonstrated that transplantation of neural stem cells expressing human nerve growth factor (NGF) improved learning and memory in rats with AD [59]. Similarly, the transplantation of NGF-expressing transduced OECs into the hippocampus restored cognitive function in rats with experimental AD [22]. Furthermore, Hu et al. reported that transplantation of brain-derived neurotrophic factor (BDNF)-expressing cholinergic-like neurons derived from human umbilical cord mesenchymal stem cells improved spatial memory in AD rats [9]. Zhang et al. reported that transplantation of BDNF-expressing bone marrow-derived mesenchymal stem cells improved cognitive function in rats with experimental AD [60]. These findings support the therapeutic potential of neurotrophin-based gene delivery strategies for the treatment of AD.

In contrast, OEC-based transplantation did not prevent pTau accumulation within the 4-week post-transplantation period. Although pTau levels showed an increasing trend and remained comparable to controls at week 8 after Aβ1–42 injection, a significant elevation was observed only at week 12. These findings suggest that tau hyperphosphorylation develops at a later stage in this acute stereotaxic Aβ1–42 model and may reflect a progressive process associated with neurodegeneration. Under pathological conditions, enhanced kinase activity and/or altered regulation of tau-related signaling pathways may contribute to tau hyperphosphorylation and subsequent tau pathology [61]. Thus, although OEC-based therapy improved cognitive performance, increased NT-3 expression, and reduced Aβ1–42 levels, it did not affect tau pathology within the limited observation period. This suggests that early cognitive deficits in this model are primarily associated with Aβ-induced synaptic dysfunction, whereas tau pathology may require longer progression or additional mechanisms to become established. Whether NT-3-expressing OECs transplantation affects pTau pathology at later stages of AD progression remains to be determined. Consistently, previous studies using an adeno-associated viral vector encoding human BDNF (AAV-BDNF) have demonstrated improved behavioral outcomes despite the absence of changes in pTau accumulation [62]. Together, these findings suggest that neurotrophin-based therapies may promote functional recovery, even when their effects on tau pathology are limited.

NT-3-expressing OEC transplantation demonstrated promising effects on cognitive function and AD-associated pathological markers. However, several limitations should be considered before clinical translation of this therapeutic approach. First, the therapeutic effects of NT-3-expressing OECs were evaluated only in an Aβ1–42-induced stereotaxic rat model of AD; therefore, validation in additional preclinical models is required. Second, the mechanisms underlying the observed reduction in brain Aβ1–42 levels remain unclear and warrant further investigation. Moreover, outcomes obtained in rodent models may not fully predict cell survival, engraftment, or long-term transgene expression in humans. To overcome this limitation, a further validation in transgenic AD model may further support translation potential. In addition, Ad5-mediated gene expression may be limited by immune responses and reduced long-term persistence; however, transgene expression in naïve rodents can persist for several months [63,64]. Alternative platforms, including helper-dependent Ad, AAV, and lentiviral vectors, may provide advantages for future clinical translation [65,66]. Finally, as olfactory dysfunction is an early manifestation of AD [67], OECs derived from patients may exhibit disease-related alterations affecting their regenerative properties, including proteomic changes, mitochondrial dysfunction, reduced neurotrophin secretion, and Aβ accumulation [68]. Future studies should therefore assess whether NT-3 transduction can restore the functional properties of patient-derived OECs for autologous application, and if it is required to establish the safety, efficacy, reproducibility, route of administration and translational feasibility of this strategy.

## 5. Conclusions

The present study demonstrates the therapeutic potential of NT-3-expressing olfactory ensheathing cells for AD treatment. Transplantation of these cells improved cognitive performance and reduced brain Aβ1–42 levels in rats with experimental AD. The therapeutic effects appear to result from the combined contribution of OEC-mediated cellular support and NT-3-mediated neurotrophic activity, highlighting the potential advantage of this dual-component strategy over approaches based solely on cell transplantation or growth factor delivery. Furthermore, the possibility of generating autologous OEC-based gene therapy products from the olfactory mucosa may facilitate the development of personalized treatments while reducing immunological risks. Although additional preclinical validation is required, these findings support further development of NT-3-expressing OECs as a promising therapeutic platform for AD.

## Figures and Tables

**Table 1:** Oligonucleotide sequences used for cloning, recombination, and sequencing.

Primer	Sequence
Fwd_*NT-3*_SalI	5′-GAGCGTCGACGCCACCATGTCCATCTTGTTTTATGTG-3′
Rev_*NT-3*_BglII	5′-TGAGCAGATCTCTTCATGTTCTTCCAATTTTTCTC-3′
Fwd_*eGFP*_HindIII	5′-GAGCAAGCTTGCCACCATGGTGAGCAAG-3′
Rev_*eGFP*_BglII	5′-TGAGCAGATCTTTACTTGTACAGCTCGTCCAT-3′

**Table 2:** Experimental groups and timeline for Alzheimer’s disease modeling and subsequent transplantation of genetically modified olfactory ensheathing cells (OECs).

Phase I: Induction (Day 0)	Phase II: Transplantation (Month 2)
Control-PBS (n = 15)	DMEM/F12 (vehicle) (n = 10)
	Euthanized for ELISA (reference) (n = 5)
Experimental-Aβ1-42 (n = 45)	DMEM/F12 (vehicle) (n = 10)
	Non-transduced OECs (0.75 × 10^6^ cells) (n = 10)
	Ad5RGD-CAG-NT-3 transduced OECs (0.75 × 10^6^ cells) (n = 10)
	Ad5RGD-CAG-EGFP transduced OECs (0.75 × 10^6^ cells) (n = 10)
	Euthanized for ELISA (reference) (n = 5)

**Table 3:** NT-3 secretion by rat OECs and A549 cells 72 h after Ad5RGD-CAG-NT-3 transduction. Data are presented as mean ± SD.

		A549	Rat OEC
NT-3, ng/mL	Non-transduced cells	0.013 ± 0.002	0.015 ± 0.001
	Ad5RGD-CAG-NT-3-transduced cells	120,531 ± 38.435	1064.398 ± 324.595

## Data Availability

The original data presented in the study are openly available in Zenodo at 10.5281/zenodo.21900684.

## Supplementary Materials

The following supporting information can be downloaded at https://www.mdpi.com/article/10.3390/cells15181706/s1. Figure S1: Generation and validation of recombinant pShuttle-CAG plasmids carrying Ntf3 and EGFP. (**a**) Screening of clones after insertion of the Ntf3 and EGFP coding sequences into the pShuttle-CAG plasmid. The expected PCR product sizes for the parental pShuttle-CAG plasmid and the plasmids containing the Ntf3 or EGFP insert are 399 bp, 1097 bp, and 1024 bp, respectively. MW—molecular weight marker. (**b**) Verification of adenoviral genome integrity within the recombinant plasmids via diagnostic restriction analysis (EcoRV and XhoI). Left: simulated restriction analysis of the plasmids in an agarose gel; right: restriction analysis of the recombinant plasmids in a 1% agarose gel. (**c**) Electrophoretic separation of plasmid fragments following digestion with the PacI restriction enzyme. Figure S2: Comparison of behavioral and biochemical parameters among control groups. (**a**) Quantification of escape latency in the Morris water maze (MWM), showing no significant differences among the Sham, Intact, and PBS groups (n = 15 rats per group). (**b**) Quantification of tissue Aβ1–42 concentration, showing no significant differences among the Sham, Intact, and PBS groups (n = 7 rats per group). (**c**) Quantification of tissue NT-3 concentration, showing no significant differences among the Sham, Intact, and PBS groups (n = 7 rats per group). Data are presented as mean ± SD; statistical analysis was performed using one-way ANOVA followed by Tukey’s post hoc test; ns: non-significant. Figure S3: In vitro transduction efficiency of Ad5RGD-CAG-EGFP in OECs and A549 cells. Merged phase-contrast and fluorescence microscopy images (excitation wavelength: 488 nm) of OECs transduced at different multiplicities of infection (MOI): (**a**) MOI = 2.5, (**b**) MOI = 7.4, (**c**) MOI = 66, and (**d**) MOI = 200. Merged phase-contrast and fluorescence microscopy images of A549 cells transduced at different MOIs: (**e**) MOI = 0.8, (**f**) MOI = 2.5, (**g**) MOI = 22, and (**h**) MOI = 66. Scale bars = 50 μm (**a**–**d**) and 40 μm (**e**–**h**). Figure S4. Survival of OECs transduced with the Ad5RGD-CAG-EGFP adenoviral vector 4 weeks after bilateral transplantation into the rat hippocampus. (**a**) Merged fluorescence image; (**b**) DAPI nuclear staining (blue); (**c**) EGFP fluorescence showing transplanted OECs (green, indicated by an arrow). Scale bar = 100 μm. Figure S5. Behavioral testing results after transplantation of various rat OEC-based preparations: time spent in the central zone in the Open Field test (week 1). Data presented as median ± IQR. Group comparisons: Kruskal–Wallis with Dunn’s post hoc test. Significance: * *p* < 0.0332, ** *p* < 0.0021, *** *p* < 0.0002, **** *p* < 0.0001.
