# Supplementary material for: NT-3-Expressing Olfactory Ensheathing Cells Reduce Brain Aβ1–42 Accumulation and Improve Cognitive Performance in a Rat Model of Alzheimer’s Disease
Source: Cells. 2026 Sep 19;15(18):1706. doi: 10.3390/cells15181706 (PMC13605328; doi:10.3390/cells15181706)
Supplement: Supplementary file 1 [file cells-15-01706-s001.zip › cells-4538660-supplementary.pdf]

Supplementary Material

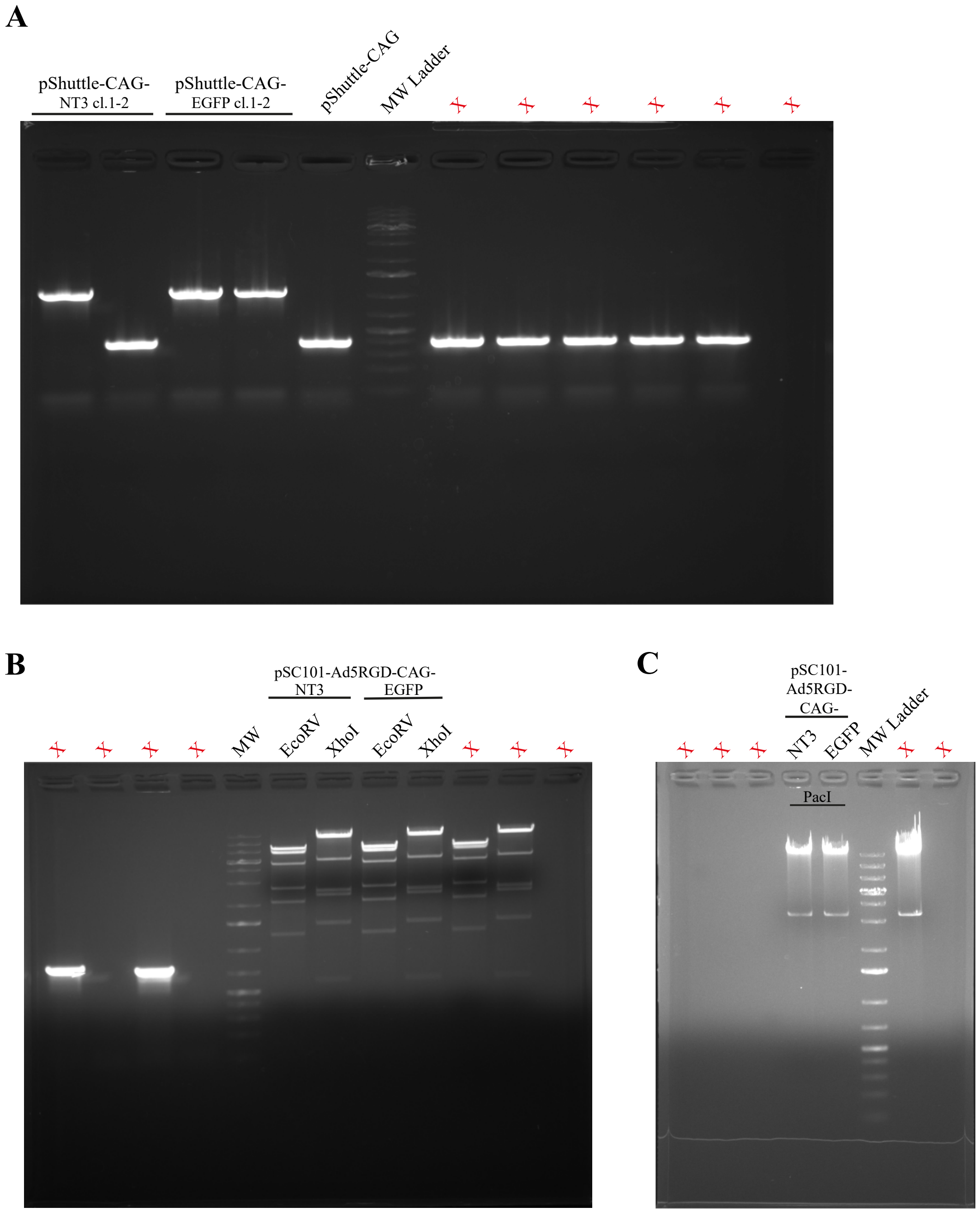

Figure S1. Generation and validation of recombinant pShuttle-CAG plasmids carrying Ntf3 and EGFP. **(a)** Screening of clones after insertion of the Ntf3 and EGFP coding sequences into the pShuttle-CAG plasmid. The expected PCR product sizes for the parental pShuttle-CAG plasmid and the plasmids containing the Ntf3 or EGFP insert are 399 bp, 1097 bp, and 1024 bp, respectively. MW –

molecular weight marker. (**b**) Verification of adenoviral genome integrity within the recombinant plasmids by diagnostic restriction analysis (EcoRV and XhoI). Left: simulated restriction analysis of the plasmids in an agarose gel; right: restriction analysis of the recombinant plasmids in a 1% agarose gel. (**c**) Electrophoretic separation of plasmid fragments following digestion with the PacI restriction enzyme.

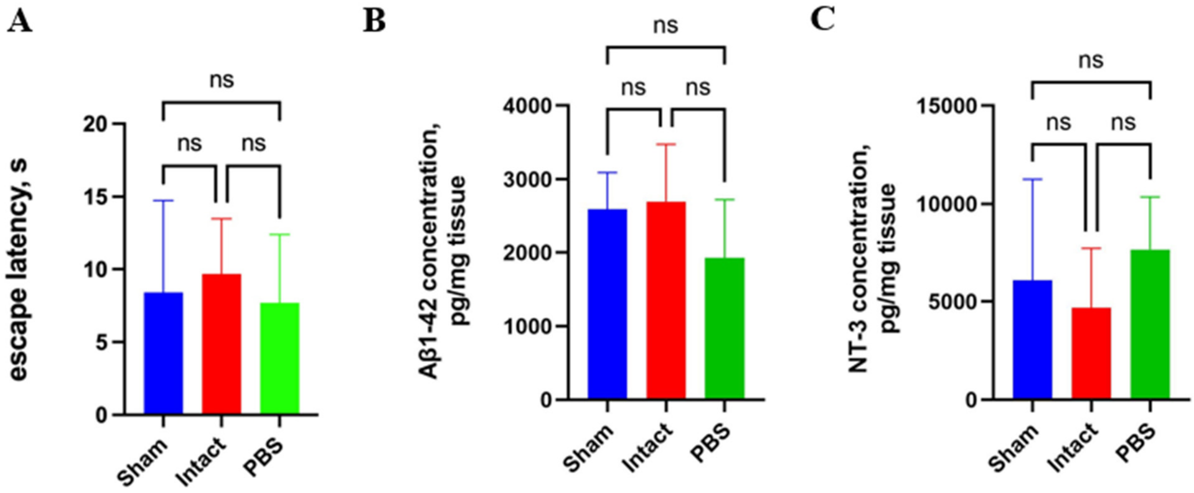

Figure S2. Comparison of behavioral and biochemical parameters among control groups. (**a**) Quantification of escape latency in the Morris water maze (MWM), showing no significant differences among the Sham, Intact, and PBS groups (n = 15 rats per group). (**b**) Quantification of tissue Aβ1–42 concentration, showing no significant differences among the Sham, Intact, and PBS groups (n = 7 rats per group). (**c**) Quantification of tissue NT-3 concentration, showing no significant differences among the Sham, Intact, and PBS groups (n = 7 rats per group). Data are presented as mean ± SD; statistical analysis was performed using one-way ANOVA followed by Tukey's post-hoc test; ns: non-significant.

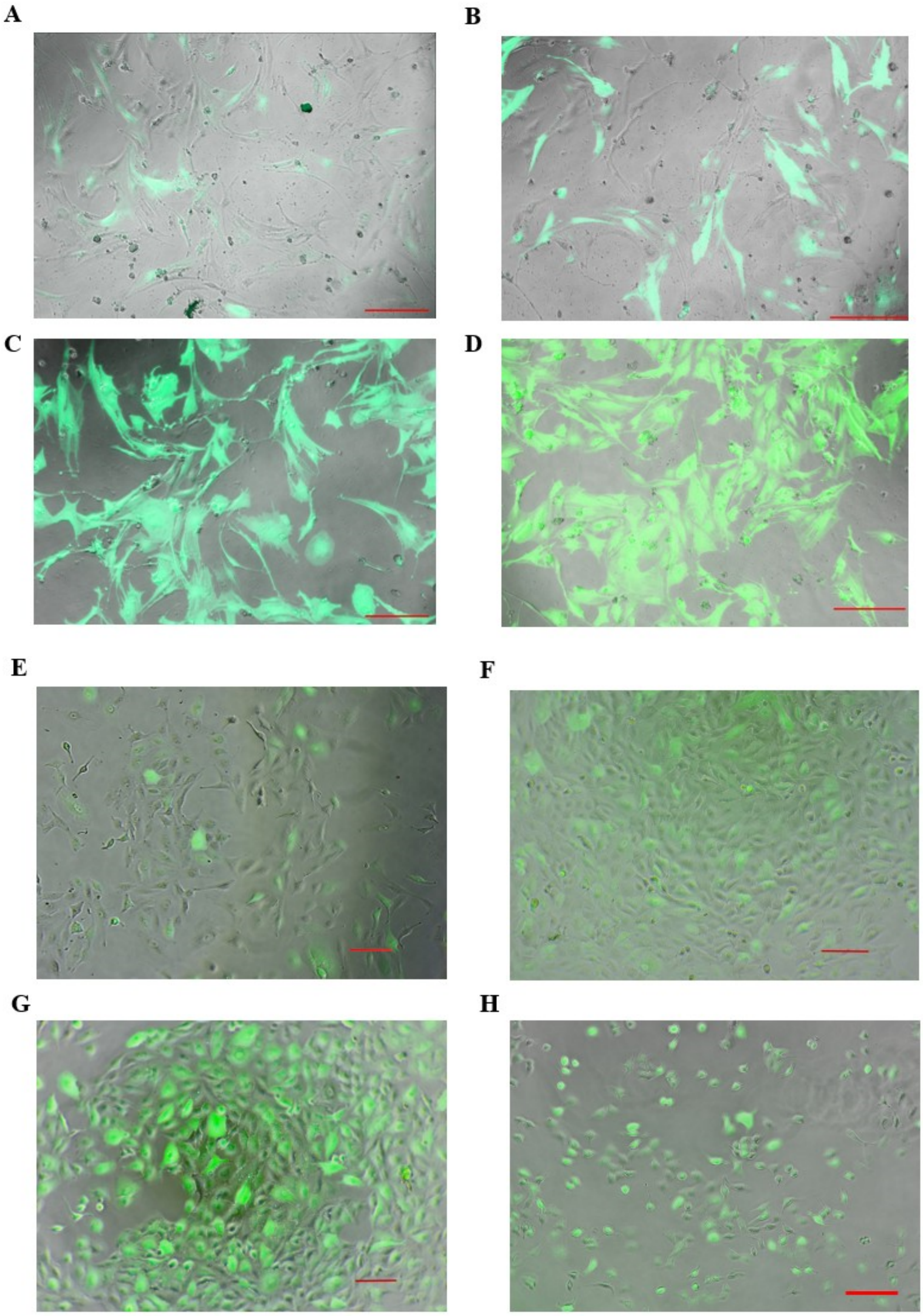

Figure S3. In vitro transduction efficiency of Ad5RGD-CAG-EGFP in (**a-d**) OECs and (**e-h**) A549 cells. Merged phase-contrast and fluorescence microscopy images (excitation wavelength: 488 nm) of OECs

transduced at different multiplicities of infection (MOI): (**a**) MOI = 2.5, (**b**) MOI = 7.4, (**c**) MOI = 66, and (**d**) MOI = 200. Merged phase-contrast and fluorescence microscopy images of A549 cells transduced at different MOIs: (**e**) MOI = 0.8, (**f**) MOI = 2.5, (**g**) MOI = 22, and (**h**) MOI = 66. Scale bars = 50 μm (**a–d**) and 40 μm (**e–h**).

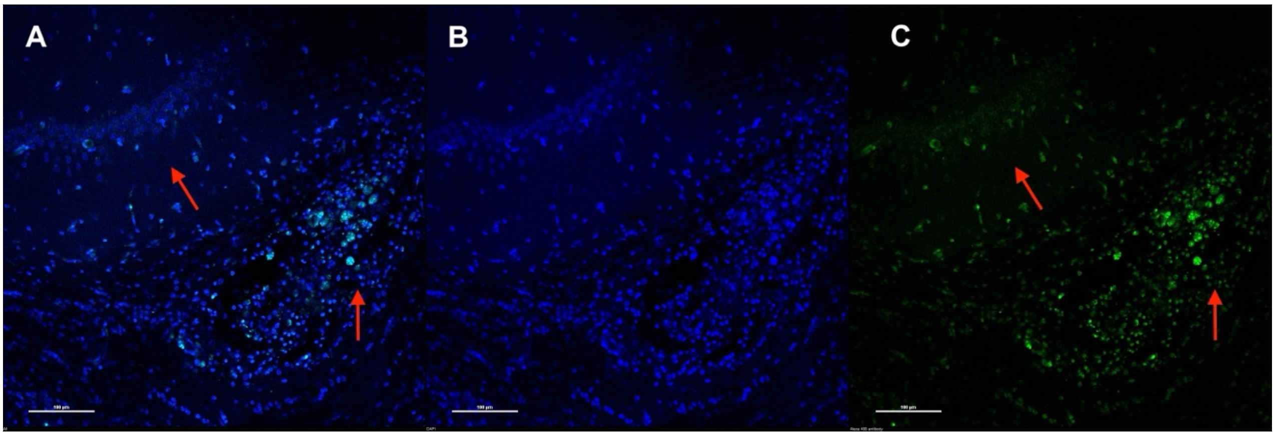

Figure S4. Survival of OECs transduced with the Ad5RGD-CAG-EGFP adenoviral vector 4 weeks after bilateral transplantation into the rat hippocampus. (**a**) Merged fluorescence image; (**b**) DAPI nuclear staining (blue); (**c**) EGFP fluorescence showing transplanted OECs (green, indicated by an arrow). Scale bar = 100 μm.

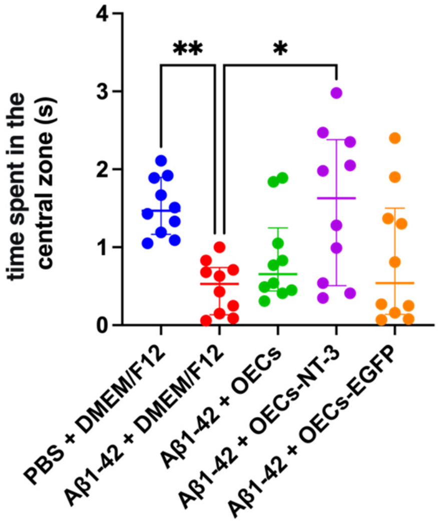

Figure S5. Behavioral testing results after transplantation of various rat OEC-based preparations: time spent in the central zone in the Open Field test (week 1). Data presented as median ± IQR. Group comparisons: Kruskal–Wallis with Dunn's post hoc test. Significance: * $p < 0.0332$, ** $p < 0.0021$, *** $p < 0.0002$, **** $p < 0.0001$.
